# BDE-99 impairs differentiation of human and mouse NPCs into the oligodendroglial lineage by species-specific modes of action

**DOI:** 10.1038/srep44861

**Published:** 2017-03-20

**Authors:** Katharina Dach, Farina Bendt, Ulrike Huebenthal, Susanne Giersiefer, Pamela J. Lein, Heike Heuer, Ellen Fritsche

**Affiliations:** 1 IUF- Leibniz Research Institute for Environmental Medicine, Auf’m Hennekamp 50, 40225, Duesseldorf, Germany; 2 Department of Molecular Biosciences, School of Veterinary Medicine, University of California, Davis, California 95616, United States

## Abstract

Polybrominated diphenyl ethers (PBDEs) are bioaccumulating flame retardants causing developmental neurotoxicity (DNT) in humans and rodents. Their DNT effects are suspected to involve thyroid hormone (TH) signaling disruption. Here, we tested the hypothesis whether disturbance of neural progenitor cell (NPC) differentiation into the oligodendrocyte lineage (O4^+^ cells) by BDE-99 involves disruption of TH action in human and mouse (h,m)NPCs. Therefore, we quantified differentiation of NPCs into O4^+^ cells and measured their maturation via expression of myelin-associated genes (h*MBP*, m*Mog*) in presence and absence of TH and/or BDE-99. T3 promoted O4^+^ cell differentiation in mouse, but not hNPCs, and induced h*MBP*/m*Mog* gene expression in both species. BDE-99 reduced generation of human and mouse O4^+^ cells, but there is no indication for BDE-99 interfering with cellular TH signaling during O4^+^ cell formation. BDE-99 reduced h*MBP* expression due to oligodendrocyte reduction, but concentrations that did not affect the number of mouse O4^+^ cells inhibited TH-induced m*Mog* transcription by a yet unknown mechanism. In addition, ascorbic acid antagonized only the BDE-99-dependent loss of human, not mouse, O4^+^ cells by a mechanism probably independent of reactive oxygen species. These data point to species-specific modes of action of BDE-99 on h/mNPC development into the oligodendrocyte lineage.

Polybrominated diphenylethers (PBDEs) were widely used as flame retardants in consumer products and although mostly banned from the markets, they remain present in the environment^1^,^2^,^3^,^4^. Main PBDE exposure sources for adults, young children, babies and fetuses comprise food, house dust, breast milk and cord blood^5^,^6^. Due to combined exposure via house dust and breast milk, toddlers present the highest PBDE body burdens of all age groups^7^,^8^,^9^. Exposure during development is of high concern as PBDEs are developmentally neurotoxic for humans^10^: high prenatal PBDE exposure correlated with lower IQs, attention deficits and hyperactivity^11^,^12^,^13^.

Thyroid hormones (THs) play a fundamental role in brain development as they guide multiple neurodevelopmental processes in rodents^14^. Specifically, TH is essential for human and rodent myelin formation^15^,^16^,^17^,^18^, which is indispensable for proper functions of neurons^19^,^20^ and the entire brain^21^.

There is increasing evidence that PBDEs might act as TH disruptors on brain development. First, PBDEs decreased TH L-thyroxine (T4) and increased thyroid stimulating hormone (TSH) blood levels in rodents^22^,^23^. And second, *in utero* PBDE-exposed animals presented similar behavioral abnormalities (hyperactivity and habituation decrease) and impaired learning^24^,^25^,^26^ as animals with propylthiouracil (PTU)-induced TH deficiency^27^. In humans, high PBDE measures were correlated with either decreased, increased or unchanged T4 and increased or decreased TSH blood levels^28^,^29^,^30^,^31^. *In vitro*, however, TH disruption of brominated diphenyl ether (BDE)−99 was suspected as this compound reduced human O4^+^ cell formation from human neural progenitor cells (hNPCs)^32^. Whether TH disruption is indeed involved in PBDE-dependent disturbance of cellular TH actions on oligodendrogenesis remains elusive.

To shed light on this question, this study aimed to elucidate if disturbance of TH signaling by BDE-99 interferes with the neurodevelopmental process of oligodendrogenesis *in vitro* and thus might be a potential mode of action (MoA) of PBDE-induced DNT. First, we characterized the effects of BDE-99 on the formation of O4^+^ cells and their maturation in human and mouse NPCs (h,mNPCs). Second, we demonstrated the functionality of TH signaling in NPCs and assessed the species-specific contribution of TH to oligodendrocyte differentiation. Third, we elucidated the species-specific MoA of BDE-99 on the formation of O4^+^ cells and their maturation in hNPCs and mNPCs.

## Results

### BDE-99 reduces NPC differentiation to the oligodendrocyte lineage

**Figure 1:**
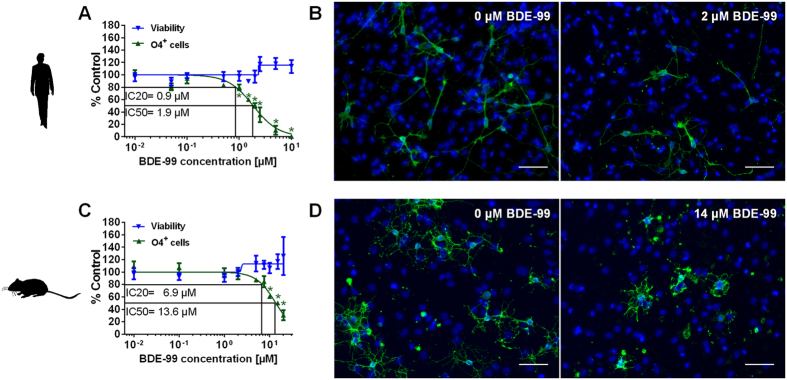
BDE-99 reduces NPC differentiation to the oligodendrocyte lineage. Human (**A**,**B**) and mouse (**C,D**) NPCs were differentiated with DMSO or increasing concentrations of BDE-99 (0.01–20 μM) for 5 days. O4^+^ cells were immunocytochemically stained with an antibody against O4 and nuclei were counterstained with Hoechst 33258. Viability was measured with the Alamar-Blue assay two hours prior fixation. (**A**,**C**) Data for viability and the percentage of O4^+^ cells/nuclei in the migration area were normalized to the solvent control (mean ± SEM, n ≥ 3). A sigmoidal curve fit was applied and for oligodendrogenesis IC_20_ and IC_50_ values were calculated. The 95% confidence intervals are 0.7–1.0 μM (human IC_20_); 1.6–2.2 μM (human IC_50_); 5.7–8.3 μM (mouse IC_20_) and 11.3–16.5 μM (mouse IC_50_). Significant differences from the DMSO control are indicates as * (p < 0.05). (**B**,**D**) Illustration of human and mouse O4^+^ cells visualized by O4 immunofluorescence stainings after treatment with DMSO or the respective IC_50_ concentration of BDE-99 after five days of differentiation (scale bar: 50 μm).

Human and mouse NPCs grown as neurospheres migrate radially out of the sphere upon plating onto a poly-D-lysin/laminin matrix and form on average 6–8% O4^+^ cells/nuclei after 5 days of migration and simultaneous differentiation^33^,^34^. These percentages are similar to the oligodendrocyte fractions in human^35^,^36^ and mouse^37^,^38^ cortex *in vivo* supporting the physiological relevance of our findings. BDE-99 reduced differentiated O4^+^ cells/nuclei of both species concentration-dependently after 5 days without reducing viability (Fig. 1A,C). Human O4^+^ cell formation was found to be 7 times more sensitive towards the BDE-99 treatment than the murine process (IC_50_: 1.9 μM and 13.6 μM BDE-99 and IC_20_: 0.9 μM and 6.9 μM for hNPCs and mNPCs, respectively) showing significance in the nanomolar range (LOEAC 900 nM). Fluorescence microscope images illustrate the BDE-99-mediated reduction in O4^+^ cells after 5 days of differentiation (Fig. 1B,D) with the human IC_50_ concentration of 2 μM not reducing number of mouse O4^+^ cells/nuclei (Supplementary Fig. S1).

### TH signaling is functional in NPCs

Human and mouse NPCs, as also previously described by others^39^,^40^, express genes encoding for TH receptors (TRs) α and β (Supplementary Fig. S2A–D). Although the expression of the gene encoding for human TRβ1 (*THRB1*) was significantly induced by the receptor active thyroid hormone L-triiodothyronine (T3), the low copy number change from 3 to 7 copies/10,000 *β-actin* makes a biological significance of this increase highly unlikely. Compared to the approximately 20-fold higher expression of TRα1 in both species it is assumed that this receptor is more important at this stage of brain development. *In vivo* TRα1 plays a role during early brain development and TRβ1expression strongly increases postnatally in the rodent brain^41^. Functionality of TRs was demonstrated by T3-induced *hairless* mRNA expression (Supplementary Fig. S2E,F). *Hairless* is a TH-inducible gene in fetal brains *in vivo*^42^,^43^ that is TRα-dependent^44^. Furthermore, T3 induced its own metabolism by stimulating *deiodinase 3* mRNA expression (Supplementary Fig. S2G,H).

### TH effects on basal and BDE-99-reduced mouse and human O4^+^ cell formation

**Figure 2:**
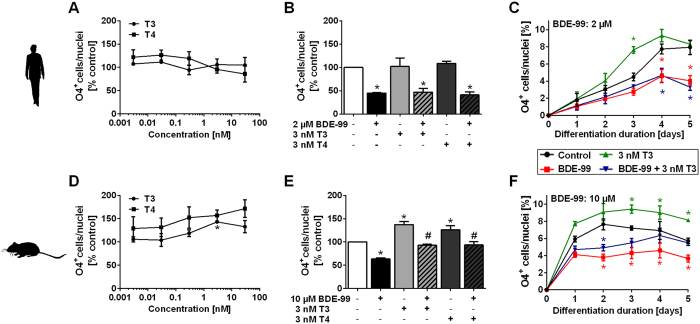
TH effects on basal and BDE-99-reduced mouse and human O4^+^ cell formation. Human (**A–C**) and mouse (**D–F**) NPCs were differentiated for 5 days (**A,B,D,E**) or 1–5 days (**C,F**) under exposure to solvent(s), BDE-99 and/or TH (T3 or T4). Immunocytochemical stainings for O4^+^ cells and counterstainings with Hoechst 33258 for nuclei were performed. (**A,D**) Data on TH (0.003–30 nM) alone are shown as mean ± SEM (n = 3 (mouse), n = 4 (human)). (**B**,**E**) Results of the co-exposure experiments with solvent, BDE-99 (approx. IC_50_ concentrations) and 3 nM T3 or T4 are presented as mean + SEM (n = 3 (human), n = 4 (mouse)) after 5 days of differentiation. (**C**,**F**) Time-course results for solvent, BDE-99 (approx. IC_50_ concentrations), 3 nM T3 and co-exposure are shown as mean ± SEM (n = 3 (human), n = 4 (mouse)). Significant differences (p < 0.05) from the solvent controls (of the respective day in **C** and **F**) are indicated as * and from BDE-99 treatment as #. The time-course curves of solvent controls (black curves in **C**,**F**) of human and mouse NPCs belonging to these experiments were previously published. This figure is not covered by the CC BY license. [*Methods Pharmacol. Toxicol.* Application of the Neurosphere Assay for DNT Hazard Assessment: Challenges and Limitations., 2016, 1–29, J. Baumann, K. Dach. M. Barenys, S. Giersiefer, J. Goniwiecha, P. J. Lein, E. Fritsche, Copyright holder: Springer Science+Business Media New York]. All rights reserved, used with permission of Springer.

T3 and T4 only induced formation of O4^+^ cells/nuclei in mNPCs, but not in hNPCs after 5 days of differentiation (Fig. 2A,D). A T3 concentration of 3 nM was used for all further experiments because it is close to the total T3 concentration in human serum^45^,^46^,^47^,^48^ and produced the highest percentage of O4^+^ cells in mNPCs (Fig. 2D). After 5 days, 3 nM T3 or T4 increased the percentage of O4^+^ cells/nuclei in control as well as in BDE-99 treated mNPCs, but not in hNPCs (Fig. 2B,E). Time-course experiments revealed that human O4^+^ cells form during the first 4 days of differentiation. Treatment of the cells with T3 accelerated, but did not increase the percentage of human O4^+^ cells after 5 days of differentiation. Moreover, T3 did not antagonize the inhibitory BDE-99 effect on human O4^+^ cell formation at any time point (Fig. 2C). In mouse cultures, O4^+^ cells formed within the first 1–2 days and T3 induced their formation in control as well as in BDE-99-treated NPCs at all timepoints with a similar potency (Fig. 2F).

### TH effects on basal and BDE-99-reduced mouse and human oligodendrocyte maturation

**Figure 3:**
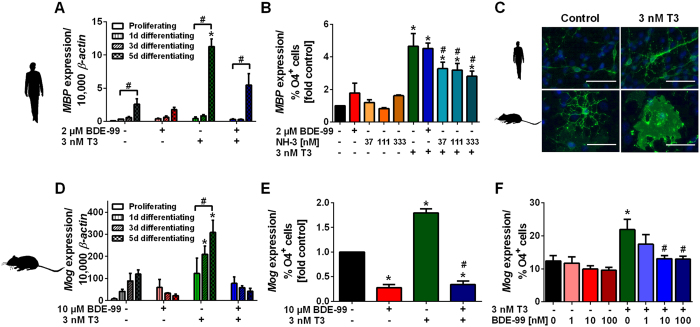
TH effects on basal and BDE-99-reduced mouse and human oligodendrocyte maturation. Human (**A–C**) and mouse (**C–F**) NPCs were differentiated with solvents, BDE-99 (approx. IC_50_ concentrations for formation of O4^+^ cells) and/or T3 for 1, 3 or 5 days (**A,D**) or 5 days (**B,C,E,F**). *MBP* (hNPCs) or *Mog* (mNPCs) expression was determined by real-time RT-PCR. Copy numbers of the genes were normalized to the expression of *β-actin*. (**A**,**D**) h*MBP* or m*Mog* expression after 1, 3 and 5 days of NPC differentiation (mean + SEM, n = 3). * Indicates significant differences (p < 0.05) from the respective solvent control and # from the same treatment at day 1. (**B**,**E**,**F**) h*MBP* or m*Mog* expression on day 5 was divided by the percentage of differentiated O4^+^ cells in the neurosphere mixed-cell migration area and then normalized to the m*Mog* expression/% O4^+^ cells of the solvent control. As positive control data of 5 days differentiated hNPCs treated with solvents, TR antagonist NH-3 and/or T3 treatment is included (**B**). Data are shown as mean + SEM (n = 3). * Indicates significant differences (p < 0.05) from the control or # from the T3 treatment. (**C**) Representative fluorescence microscope pictures of 5 days differentiated human and mouse O4^+^ cells stained with antibody against O4 in presence of solvent or 3 nM T3 (scale bar: 50 μm).

For investigating oligodendrocyte maturation, changes in expression of the oligodendrocyte-specific markers human myelin basic protein (h*MBP*) and murine myelin oligodendrocyte glycoprotein (m*Mog*) were analyzed in the total neurosphere cell population over a time-course of 5 days (Fig. 3A,D). These different markers were used, because human oligodendrocytes do not express the late marker *MOG* after 5 days of differentiation due to their immaturity, while for murine oligodendrocytes - due to their high maturation stage - *Mbp* was already highly expressed on day 1 of differentiation and did not increase over differentiation time (data not shown). We also confirmed this species-dependent difference in oligodendrocyte maturation stage by morphological analysis as branched, sheet-forming O4^+^ cells found in murine culture represent phenotypically a more mature state than the more linear O4^+^ cells observed in human cultures (Fig. 3C). Both, h*MBP* and m*Mog* expression increased significantly during the 5 days of differentiation indicating oligodendrocyte maturation (Fig. 3A,D). During this time period, T3 significantly induced gene copy numbers of NPCs’ h*MBP* and m*Mog*, while BDE-99 reduced T3-induced h*MBP* as well as m*Mog* expression. To evaluate if this reduced h*MBP* and m*Mog* expression is a consequence of reduced O4^+^ cell formation or rather a consequence of compromised oligodendrocyte maturation, h*MBP* and m*Mog* expression after 5 days of differentiation were normalized to the percentage of O4^+^ cells in the respective cultures (6–8% O4^+^ cells; Fig. 3B,E) by calculation of the maturation quotient (Q_M_):


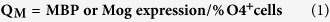


Q_M_ clearly revealed that 3 nM T3 significantly increased the maturation stage of oligodendrocytes in both species (human: 4.4-fold, mouse: 1.8-fold). Treatment of hNPCs with 2 μM BDE-99 (IC_50_ concentration for inhibition of human O4^+^ cell formation, Fig. 1) did not reduce human Q_M_, neither alone nor in combination with T3 (Fig. 3B), showing that the BDE-99-dependent reduction in h*MBP* expression was solely due to the lower number of O4^+^ cells, and thus TH-independent, while the TR antagonist NH-3 reduced Q_M_ without affecting cell viability (Supplementary Fig. S4) supporting a role for TR in T3-induced h*MBP* expression. In contrast, 10 μM BDE-99 (approx. IC_50_ inhibition of murine O4^+^ cell formation; 14 μM BDE-99 are not medium-soluble with a DMSO concentration of 0.1%, Fig. 1) given alone or in combination with T3 reduced murine Q_M_ to 30% of the respective solvent control value (Fig. 3E) suggesting a TH-independent effect at this concentration. BDE-99 concentrations, however, that alone did not affect oligodendrocyte maturation (1–100 nM), significantly lowered T3-induced murine Q_M_. The underlying molecular mechanism needs further elucidation (Fig. 3F).

### TR involvement in TH and BDE-99 effects on mNPCs’ differentiation to O4^+^ cells and mMog gene expression

**Figure 4:**
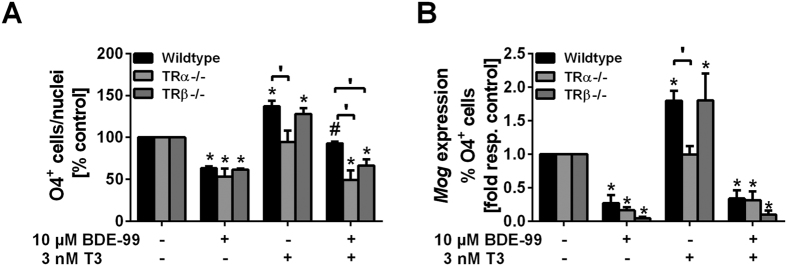
TR involvement in TH and BDE-99 effects on mNPCs’ differentiation into O4^+^ cells and m*Mog* gene expression. Neurospheres prepared from wildtype, TRα−/− and TRβ−/− mouse brains were differentiated for 5 days in the presence of solvents, 10 μM BDE-99 and/or T3. (**A**) Immunocytochemical stainings of O4^+^ cells were performed and nuclei were counterstained with Hoechst 33258. The percentages of O4^+^-cells/nuclei are shown normalized to the respective genotype solvent controls (absolute percentage of O4^+^ cells does not differ between solvent treated wildtype and TR knockout neurospheres; Supplementary Fig. S5) (mean + SEM, n = 4). (**B**) m*Mog* copy numbers determined by real time RT-PCR were first normalized to the *β-actin* expression, then divided by the percentage of O4^+^ cells and finally normalized to the m*Mog* expression/% O4^+^ cells of the respective solvent control (maturation in solvent treated samples of wildtype and knockout mice did not differ). Data are shown as mean + SEM, n = 3 (wildtype), n = 4 (knockout). p < 0.05 was considered as significantly different from the respective solvent control (*), BDE-99 treatment (#) or the respective wildtype treatment (′).

The involvement of TRs in T3 and BDE-99 effects on mNPCs’ differentiation to O4^+^ cells and m*Mog* gene expression was studied using neurosphere cultures prepared from PND1 TRα and β knockout mouse brains. Number of O4^+^ cells/nuclei as well as m*Mog* gene expression/% O4^+^ cells (Q_M_) were normalized to the respective genotype control because raw values did not differ significantly between genotype controls (Supplementary Fig. S5). T3 significantly induced number of O4^+^ cells/nuclei (Fig. 4A) and their maturation (Fig. 4B) in wildtype and TRβ, but not in TRα knockout neurospheres, confirming the specific involvement of TRα in T3-induced O4^+^ cell formation and maturation. On the contrary, BDE-99 effects on mouse O4^+^ cell formation and oligodendrocyte maturation were not mediated by TRs, because 10 μM BDE-99 reduced the number of differentiated O4^+^ cells as well as *Mog* expression/% O4^+^ cells in TR knockout neurospheres comparable with wildtype neurospheres (Fig. 4A,B).

To confirm that 10 μM BDE-99 indeed does not interfere with TR-mediated TH signaling, expression of *TRs, deiodinase 3* and *hairless*, the latter TR-dependent gene products, was evaluated. BDE-99 did not influence *TR* expression (Supplementary Fig. S2A–D) or TRα-mediated transcription of *hairless* alone or in combination with T3 (Supplementary Fig. S2E,F). Furthermore, BDE-99 did not alter basal or T3-induced *deiodinase 3* expression (Supplementary Fig. S2G,H).

### Reactive Oxygen Species (ROS) do not seem to be involved in BDE-99-dependent reduction of O4^+^ cells

**Figure 5:**
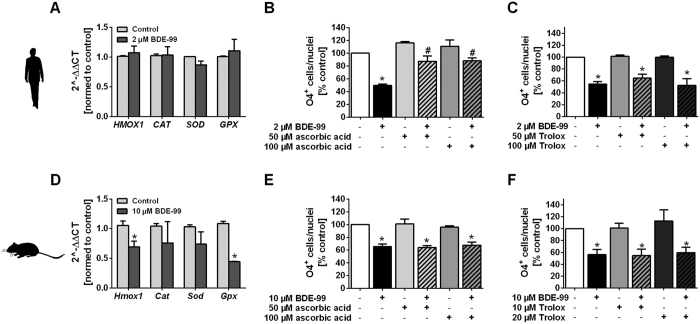
Reactive Oxygen Species (ROS) do not seem to be involved in BDE-99-dependent reduction of O4^+^ cells. Human (**A–C**) and mouse (**D–F**) NPCs were differentiated for 24 h (**A**,**D**) or 5 days (**B**,**C**,**E**,**F**) in presence of solvent, BDE-99 and/or antioxidant (ascorbic acid or Trolox). Immunocytochemical O4 stainings (**B**,**C**,**E**,**F**) or gene expression studies by real-time RT-PCR (**A**,**D**) were performed. (**A**,**D**) Expression of the ROS-related genes *heme oxygenase-1 (HMOX-1/Hmox-1), catalase (CAT/Cat), superoxide dismutase (SOD/Sod*) and *glutathione peroxidase (GPX/Gpx*) was evaluated with the ΔΔCT method (housekeeping gene: *β-actin*; gene expression normalized to the expression in solvent sample). Results are shown as 2^−ΔΔCT^ (mean + SEM, n = 3). (**B**,**C**,**E**,**F**) Percentage of O4^+^ cells/nuclei normalized to the solvent control (mean + SEM, n = 3–4). p < 0.05 was considered significantly different from solvent control (*) or from BDE-99 treatment (#).

Since our studies revealed that TH disruption was not the mechanism by which BDE-99 compromised formation of human O4^+^ cells (Fig. 2B,C), we considered an alternative BDE-99 MoA, i.e. ROS formation. We hypothesized that the production of ROS might be involved in BDE-99-induced toxicity on O4^+^ cell formation. One indicator of ROS production is an adaptive induction of antioxidative defense gene expression like *heme oxygenase 1, catalase, superoxide dismutase* and *glutathione peroxidase*. 2 μM BDE-99 did not alter the expression of any of these genes after 24 hours of BDE-99 exposure suggesting that BDE-99 is not producing an excess of ROS in hNPCs (Fig. 5A). However, application of ascorbic acid (50 and 100 μM, not inducing O4^+^ cell formation *per se*) antagonized the BDE-99-dependent reduction of human O4^+^ cell formation (Fig. 5B). As Trolox, a water-soluble derivative of vitamin E and effective ROS-scavenger, did not exert comparable antagonizing effects in presence of BDE-99 (Fig. 5C), it is likely that ascorbic acid rescues BDE-99-reduced O4^+^ cell formation by a mechanism other than ROS scavenging. In contrast to human O4^+^ cell formation, mouse O4^+^ cell formation was not rescued by ascorbic acid against BDE-99 (Fig. 5E). Also Trolox did not rescue murine O4^+^ cells against BDE-99-induced toxicity (Fig. 5F). It is to note, that for scavenging BDE-99 effects in mNPCs, lower Trolox concentrations (10 and 20 μM) were used than for hNPCs since in mNPCs, 50 and 100 μM Trolox reduced the culture viability and O4^+^ cell formation (data not shown).

## Discussion

PBDEs were recently classified as human DNT compounds^10^. The molecular mechanisms of PBDE-induced DNT, however, remain elusive. PBDEs alter TH homeostasis in rodents and interfere with TH signaling *in vitro*^49^. In addition, BDE-99 reduced oligodendrocyte differentiation of human NPCs^32^. Because TH is crucial for oligodendrogenesis and/or myelin production *in vivo*^17^,^18^,^19^,^50^,^51^,^52^, we investigated in this study if disruption of cellular TH signaling in hNPCs and mNPCs might be an underlying molecular mechanism for BDE-99-mediated reduction of O4^+^ cell formation and oligodendrocyte maturation.

BDE-99 reduced human and murine O4^+^ cell formation in a concentration-dependent manner (IC_50_ 1.9 μM and 13.6 μM, respectively and IC_20_ 0.9 μM and 6.9 μM, respectively) with human NPCs being 7-times more sensitive towards BDE-99 exposure than their murine counterparts. These data are in accordance with two other publications dealing with PBDE effects on oligodendrogenesis: our previous study with an IC_50_ for inhibiting human O4^+^ cell formation around 1 μM BDE-99^32^ and inhibition of murine NSC differentiation into O4^+^ cells with an IC_25_ for BDE-47 of 10 μM^53^.

The IC_20_ value for inhibiting human O4^+^ cell formation identified in this study lies in the upper nanomolar range (900 nM; approximately 450 ng/ml). Breast milk fed children are assumed to reach a daily PBDE exposure up to 306 ng/kg/day^54^,^55^ and the serum concentration of the five most abundant PBDEs reached 480 ng/g lipid weight (lw; approx. 960 nM in serum lipids) for Californian toddlers^7^. *In vivo*, up to a 1.5-fold accumulation of PBDEs in brain lipids compared to the corresponding serum lipid samples in birds^56^ and a 5.5 fold accumulation of BDE-47 in the rat brain versus serum^57^ were observed indicating that PBDEs pass the blood-brain barrier and accumulate in the brain. Assuming similar accumulation in human brain lipids, our rough kinetic estimation suggests that toddler PBDE brain lipid concentrations could exceed 720 ng/g lw (approx. 1440 nM in brain lipids), which is higher than the observed IC_20_ (900 nM) for human NPC differentiation in this study.

To test the hypothesis if TH disruption might be the underlying molecular mechanism for BDE-99-dependent inhibition of O4^+^ cell differentiation, we first characterized TH effects on human and mouse oligodendrogenesis. TH alone accelerated, but did not induce O4^+^ cell formation from hNPCs, while it induced the number of O4^+^ cells differentiated from mNPCs. In addition, TH enhanced human and murine oligodendrocyte maturation. Accelerated oligodendrocyte maturation due to TH treatment was also observed in mouse and human oligodendrocyte precursor cells (OPCs)^58^ and in embryonic hNPCs differentiating into O4^+^ cells *in vitro*^59^ supporting our findings. Moreover, similar to the rodent *in vitro* findings in this study, TH determines timing and amount of oligodendrocyte formation in rodents^52^,^60^,^61^,^62^ in a TRα-dependent manner^63^,^64^, and hypothyroid mice express lower levels of *Mbp* than euthyroid mice, which might be due to less oligodendrocyte formation, their reduced maturation or a combination of both^52^. Neurospheres prepared from TRα and TRβ knockout mice indicated that TH-dependent induction of murine O4^+^ cell formation and oligodendrocyte maturation from mNPCs *in vitro* was also TRα-, not TRβ-dependent. Human patients with hypothyroidism during brain development also show delayed myelination^15^,^16^,^65^ due to less oligodendrocyte formation or maturation or a combination of both^66^. Our species-comparative *in vitro* study suggests that in contrast to mNPCs, TH mainly guides hNPC-derived oligodendrocyte maturation as supported by others^58^,^59^. Concerning the speed of O4^+^ cell formation and oligodendrocyte maturation we found that these processes happen much faster in mouse than in human NPC cultures. Also *in vivo* oligodendrocytes form and mature much faster in rodents than in humans^67^. Thus, the closeness of the *in vitro* systems mirroring species-specific maturation speed strengthens the physiological relevance of our cell systems.

BDE-99 reduced NPC differentiation into O4^+^ cells in both species independent of TH as (i) human O4^+^ cell formation is not affected by TH and the TR antagonist NH-3 does not affect human NPC differentiation into O4^+^ cells (Supplementary Fig. S4) and (ii) BDE-99 reduces number of mouse O4^+^ cells in TH-induced neurospheres with the same magnitude than in untreated cells. Experiments with TRα and TRβ knockout mouse neurospheres support this data and add the information that TRs are not involved in BDE-99 action on O4^+^ cell formation. In accordance, BDE-99 did not inhibit T3-induced expression of *hairless*, a gene that is regulated by TRα in mice^44^ and did not alter TR or T3-induced *deiodinase 3* mRNA expression in either species.

As another possible MoA we postulated that the formation of ROS might be involved in BDE-99 toxicity on oligodendrogenesis. ROS formation by PBDEs is discussed controversially in the literature depending on the congener, cell type and method used^49^. BDE-99 did not up-regulate ROS-related genes in hNPCs and mNPCs and sub-cytotoxic concentrations of the antioxidant Trolox did not antagonize the BDE-99 effect on O4^+^ cell formation. Interestingly, ascorbic acid (50–100 μM) rescued BDE-99-reduced O4^+^ cell formation in human, but not in mouse NPCs at concentrations not inducing oligodendrocytes in the cultures *per se*. A high ascorbic acid concentration (500 μM) strongly induced formation of human, but not mouse O4^+^ cells (Supplementary Fig. S6). Ascorbic acid enables hydroxylation of proline to hydroxyproline^68^, a major component of the extracellular matrix protein collagen promoting rodent myelination of dorsal root ganglion neurons by Schwann cells in the peripheral nervous system^69^,^70^,^71^. The effects of ascorbic acid on central nervous system oligodendrogenesis are not known. However, the mechanism by which ascorbic acid antagonizes BDE-99-dependent reduction in human O4^+^ cell formation might involve collagen production. Interference of BDE-99 with mouse O4^+^ cell formation is clearly mediated by a different, yet unknown, MoA, as neither ascorbic acid nor Trolox antagonize this adverse effect.

The maturation quotient Q_M_ demonstrated that BDE-99 at the IC_50_ concentration for O4^+^ cell formation did not affect human, but inhibited mouse gene expression related to oligodendrocyte maturation. These BDE-99 effects on m*Mog* expression were also independent of TH signaling as TH did not antagonize the effect and TR knockout neurospheres were not protected against BDE-99-dependent loss of O4^+^ cells. Moreover, ascorbic acid did not antagonize reduction of BDE-99-dependent m*Mog* expression (Supplementary Fig. S7) pointing to a mechanism distinct from the MoA in hNPCs. BDE-99 concentrations, which did not interfere with basal O4^+^ cell differentiation or m*Mog* expression, however, prohibited T3 induction of m*Mog* by a yet unknown mechanism.

Another possibility that was not addressed within this paper but is worth exploring in the future is that BDE-99 does not directly act on oligodendrocytes, but exerts its actions indirectly e.g. via astrocyte signaling. Astrocytes play a major role in growth factor release like PDGF, FGF2, LIF, CNTF, IGF-1, NT-3, extracellular matrix-related molecules or BMPs, which obtain known functions in oligodendrogenesis^19^,^72^,^73^,^74^. These soluble factors have the ability to influence oligodendrocyte differentiation e.g. by epigenetic mechanisms and thus might provide a speculative rationale for BDE-99-dependent effects on oligodendrocyte differentiation via suppressed gene expression in cells of the oligodendrocyte lineage^72^. Unique features of the ‘Neurosphere Assay’ allowing investigations of such paracrine aspects in the future are its multicellularity, and the possibility to study multiple species in equivalent cell systems. However, in case of e.g. RT-PCR evaluation of cell type-specific markers the desired cell population might only be a fraction of the total cell number like in this study, where we find 6–8% oligodendrocytes after 5 days of differentiation^34^. With regards to the species comparison, the timing aspect has to be carefully considered as speed of developmental processes observed in neurospheres seem to reflect at least to some extent the pace of the species of cell origin, which is very different between humans and rodents^33^.

## Conclusions

We developed a human and mouse NPC-based test system to identify compounds with adverse effects on oligodendrogenesis including TH signaling disruptors. With this test we identified BDE-99 as an inhibitor of O4^+^ cell differentiation in both species and a blocker of oligodendrocyte maturation-related gene expression (m*Mog*) only in mouse cultures. BDE-99 effects follow unknown MoAs in both species, but ascorbic acid rescued inhibited O4^+^ cell differentiation only in human cultures, while it exerted no effect on any of the endpoints studied in mouse neurospheres. This study indicates the need to consider species differences in susceptibility and MoA analyses of chemicals for their DNT potential and thus suggests the necessity of using human cells for human hazard characterization of compounds that might be toxic for the developing brain.

## Methods

### Chemicals

Brominated diphenyl ether (BDE)-99 was kindly provided by U. Strähle from the Karlsruhe Institute of Technology, thyroid hormones (THs) L-3,3′,5- triiodothyronine (T3) and L-3,3′,5,5′- tetraiodothyronine (T4), L-ascorbic acid and Trolox (vitamin E analog) were purchased from Sigma Aldrich. Chemicals were dissolved as follows: 1, 2, 6.66, 10 mM stocks of BDE-99 in dimethyl sulfoxide (DMSO; Carl Roth GmbH), 300 μM T3 or T4 in a 1:1 dilution of 96% ethanol and 1 M HCl (both Carl Roth GmbH) (‘EtOH/HCl’), 100 mM ascorbic acid in H_2_O, 200 mM Trolox in DMSO. TR antagonist NH-3^75^ was diluted in DMSO and a 1 mM stock was prepared. Solvent concentrations used in the experiments were 0.1% (co-treatment experiments) or 0.3% (dose-response experiments) DMSO for BDE-99, 0.1% DMSO for NH-3, 0.01% EtOH/HCl (T3, T4), 0.05% DMSO (Trolox), 0.1% or 0.5% H_2_O (L-ascorbic acid) or the respective combination in the co-treatments. For antagonization experiments, IC_50_ concentrations for BDE-99 (2 μM and 10 μM (approx.) for hNPCs and mNPCs, respectively − 14 μM BDE-99 are not medium-soluble with a DMSO concentration of 0.1%) were chosen.

### Neurosphere Culture

Human neural progenitor cells (gestational week (GW) 16–20) were purchased from Lonza Verviers SPRL (Belgium). Developmental time-matched^76^ mouse NPCs from postnatal day (PND) 1 were prepared as described previously for rat NPCs^77^ with the modification that only the mouse forebrain was used and digested with papain for 7 min. Experiments with human NPCs purchased from Lonza Verviers SPRL (Belgium) are ethical approved by the ethics committee of the Heinrich-Heine-University, Düsseldorf. Preparation of NPCs from mouse brain tissue and experiments with mouse NPCs are in accordance with German regulations and the experimental guidelines of the State Agency for Nature, Environment and Consumer Protection in North Rhine-Westphalia in Germany (LANUV) and are approved by the LANUV (license number: 84–02.05.40.14.140). To study the involvement of TH receptors (TRs) in oligodendrogenesis, NPCs from TRα and TRβ knockout mice were prepared. Human and mouse NPCs were cultured as neurospheres in proliferation medium consisting of DMEM (Life Technologies) and Hams F12 (Life Technologies) (3:1) supplemented with 2% B27 (Life Technologies), 20 ng/ml epidermal growth factor (EGF, Life Technologies), 20 ng/mL (hNPCs) or 10 ng/ml (mNPCs) recombinant human fibroblast growth factor (FGF, R&D Systems), and 1% penicillin and streptomycin (Pan-Biotech). Neurospheres were maintained at 37 °C with 5% CO_2_ and passaged mechanically with a tissue chopper to 0.2 mm once a week. Half of the medium was replaced thrice a week.

### Differentiation of NPCs

NPCs were chopped to 0.2 mm 1–3 days (hNPCs) or 3–5 days (mNPCs) before plating. The longer time between chopping and plating is due to the fact that mouse neurospheres need longer to regenerate and form even, round spheres again. Human or mouse NPCs were differentiated in differentiation medium [DMEM (Life Technologies), Hams F12 (Life Technologies) 3:1 supplemented with 1% of N2 (Life Technologies) and 1% penicillin and streptomycin (Pan-Biotech) and only for the mouse 1% hormone-free fetal calf serum (FCS; Biochrom) to prevent apoptosis was added] containing the respective exposure(s) or solvent(s). Half of the exposure/solvent medium was refreshed at day 2 of differentiation. Under differentiating conditions NPCs radially migrate out of the neurosphere core (migration area) and differentiate into the three major cell types of the brain: neurons, oligodendrocytes and astrocytes^78^,^79^.

#### Differentiation of NPCs for viability measurement and staining of O4^+^ cells

Neurospheres of 0.3 mm diameter were washed in differentiation medium and plated into poly-D-lysine (PDL)/laminin (Sigma Aldrich) coated 8-chamber glass cover slides (LMS Consult). In each chamber, five neurospheres were plated in 500 μl differentiation medium containing the respective exposure(s) or solvent(s). Neurospheres were differentiated for 5 days or in the time course experiment for 1, 2, 3, 4 and 5 days, where 5 slides were plated on the same day and one was fixed each day. Each experiment was repeated at least 3 times. Mean and SEM were calculated from those repeated experiments.

#### Differentiation of NPCs for RT-PCR

Neurospheres were differentiated in PDL/laminin coated wells of 24 well plates (Sarstedt) in 1 ml differentiation medium containing the respective exposure(s)/solvent(s). Three wells with ten 0.3 mm neurospheres each were used per exposure condition. Neurospheres were differentiated for 5 days or in time course experiments for 1, 3 and 5 days, where 3 plates were plated at the same day and cells in one plate were lysed on each day 1, 3 or 5 of differentiation. Each experiment was repeated at least 3 times. Mean and SEM were calculated from those repeated experiments.

### Viability assay

Cell viability was assessed using Alamar Blue reagent (CellTiter-Blue assay, Promega) by measuring mitochondrial reductase activity after 5 days of differentiation in the same slides which cells were later fixed and immunocytochemically stained for O4^+^ cells. The reagent was pre-diluted 1:3 in differentiation medium and added to the chambers (1:4) two hours prior to fixation. The neurospheres were incubated for 2 h at 37 °C and 5% CO_2_. The incubated reagent was pipetted into a 96-well plate in technical duplicates of 100 μl to determine fluorescence with the Tecan infinite M200 Pro reader (ex: 540 nm; em: 590 nm). Background of only medium without cells was subtracted from the sample RFU values. Duplicate RFU values from each chamber were pooled. Each viability experiment was repeated at least three times independently. Mean and SEM were calculated from those repeated experiments. Viability data which are not included in the main manuscript are shown in the Supplementary Fig. S3.

### Immunocytochemistry

After the respective differentiation times (and viability measurement) neurospheres were fixed with 4% paraformaldehyde (PFA, Merck) at 37 °C for 30 min and stored in phosphate buffered saline (PBS; Biochrom) at 4 °C until the immunocytochemical staining for O4 was performed. Cells were stained overnight with 30 μl first antibody solution (1:200 mouse IgM anti-O4 antibody (R&D Systems), 10% goat serum (Sigma Aldrich) in PBS) per chamber. After three 5 min washing steps in PBS 30 μl secondary antibody solution (1:250 Alexa Fluor 488 anti-mouse IgM (Life Technologies), 2% Hoechst 33258, 1% goat serum in PBS) were added per chamber and cells were stained for 30 min at 37 °C. After three additional 5 min washing steps in PBS, cells were washed once in distilled water and then the slide was mounted with a glass cover slip by using AquaPolyMount (Polysciences).

### Fluorescence microscopy and quantification of O4^+^ cells

In each experiment five neurospheres/treatment and two fluorescence images/neurosphere, taken at defined, opposite sides of the neurosphere core (in the same distance to the neurosphere core for each differentiation day), were evaluated resulting in 10 image evaluations/treatment/experiment. Images were taken using two channels of a fluorescent microscope (Zeiss, Axio Observer D1): ex: 359 nm; em: 461 nm for visualizing Hoechst staining and ex: 495 nm; em: 519 nm for O4 evaluation. Images were converted to 8-bit, binarized and watershed transformed for quantification of the number of nuclei by using an ImageJ macro. Only segments satisfying suitable size constraints (x-y) were considered as valid nuclei. O4^+^ cells were counted manually. Then the percentage of O4^+^ cells/nuclei was calculated for each picture and the percentages were pooled between the two pictures of the same neurosphere. Afterwards the mean and standard deviation between the five neurospheres was calculated for each treatment. The solvent control was set to 100% (except for the time-course experiment showing formation of O4^+^ cells over time). Each figure contains data from at least three independent experiments and for each condition mean and SEM were calculated from those independent experiments.

On average for all O4^+^ cell formation experiments included in the manuscript, solvent controls of hNPCs contained 620 nuclei and 38 O4^+^ cells per image (6% O4^+^ cells) and of mNPCs with a less dense migration area 310 nuclei and 18 O4^+^ cells (6% O4^+^ cells) per image. For ten microscope pictures per solvent control this ends up in approximately 380 O4^+^ cells/6200 nuclei for hNPCs and 180 O4^+^ cells/3100 nuclei for mNPCs per n. Substance treatment does not change the amount of nuclei per picture, but only the amount of O4^+^ cells.

### Quantitative RT-PCR (qRT-PCR)

RNA of untreated proliferating (triplicated samples of 50 neurospheres with a 0.3 mm diameter were collected 2 (hNPCs) or 3 (mNPCs) days after chopping) or 1,3 or 5 days under exposures/solvents differentiated NPCs (as described above) was isolated (RNeasy Mini Kit, Qiagen, Hilden, Germany) and cDNA was transcribed (Quantitect Reverse Transcription Kit, Qiagen) according to the manufacturer instructions. qRT-PCR was performed using QuantiFast SYBR Green PCR Kit (Qiagen) in the Rotor Gene Q cycler (Qiagen). Copy numbers of the gene of interest were normalized to *β-actin* expression by using standards or expression was evaluated by the ddCT method normalizing to *β-actin* expression in the solvent control samples. Each PCR experiment was performed at least three times independently. Means and SEMs were calculated from those independent experiments. The detailed PCR protocol and details for evaluation as well as the used primer sequences are given in the Supplementary Material.

### Statistics

For dose-response curves a sigmoidal curve fit (variable slope) was applied using Graphpad Prism 6.0. Data was analyzed with the same software using OneWay ANOVA for dose-response experiments, TwoWay ANOVA for co-exposure and time-course experiments (non-repeated measures) or a student’s t-test for comparison of wildtype and knockout mice within the same treatment. As post-hoc tests Bonferroni’s (OneWay ANOVA) or Tukey’s (TwoWay ANOVA) multiple comparison test were used. T-tests were performed with Welsh’s correction assuming different standard deviations for the treatments. Significance threshold was established at p < 0.05.

## Additional Information

**How to cite this article:** Dach, K. *et al*. BDE-99 impairs differentiation of human and mouse NPCs into the oligodendroglial lineage by species-specific modes of action. *Sci. Rep.* **7**, 44861; doi: 10.1038/srep44861 (2017).

**Publisher's note:** Springer Nature remains neutral with regard to jurisdictional claims in published maps and institutional affiliations.

## Supplementary Material

Supplementary Material
