# Supplementary material for: BDE-99 impairs differentiation of human and mouse NPCs into the oligodendroglial lineage by species-specific modes of action
Source: Sci Rep. 2017 Mar 20;7:44861. doi: 10.1038/srep44861 (PMC5357893; doi:10.1038/srep44861)
Supplement: Supplementary Material [file srep44861-s1.pdf]

SUPPLEMENTARY MATERIAL

BDE-99 impairs differentiation of human and mouse NPC into the oligodendroglial lineage by species-specific modes of actions

Katharina Dach[1], Farina Bendt[1], Ulrike Huebenthal[1], Susanne Giersiefer[1], Pamela J. Lein[2], Heike Heuer[1] and Ellen Fritsche[1]*

PCR procedure/standards/ddCT....................2

Fig. S1: Fluorescence microscope images of mouse O4+ cells differentiated with BDE-99 treatment....................4

Fig. S2: Expression and functionality of TH signaling component....................5

Fig. S3: Viability data for substance treatments in human, mouse wildtype and mouse TR knockout NPC experiments....................7

Fig. S4: Viability and formation of O4+ cells of NH-3 and/or T3 treated hNPCs....................8

Fig. S5: Solvent control data for wildtype, TRα-/- and TRβ-/- mouse NPCs for oligodendrocyte formation and maturation.................... 9

Fig. S6: Ascorbic acid effects on formation and maturation of O4+ cells from human and mouse NPCs…....................10

Fig. S7: m*Mog* expression after mNPC treatment with BDE-99 and/or ascorbic acid.....11

Table 1: Primer sequences....................12

PCR procedure/standards/ddCT

The PCR mix consisted of 7.5 µl of PCR Master Mix SybrFAST (Qiagen, Hilden, Germany), 2.5 µl solutions of each primer (stock concentration 4 µM) and 2.5 µl of cDNA (1:2.5 diluted). The application started with an initial incubation step of 7 min at 95°C to activate the DNA polymerase. The conditions for PCR amplifications were 47 cycles of 10 sec at 95°C for denaturation, 35 sec at 60 °C for primer annealing, elongation and fluorescence detection. We evaluated gene expression using the cycle threshold (Ct) value from each sample. The maximum number of cycles accepted were: 9-15 (*β-actin*); 12-25 (genes of interest); 25-n.d. (negative control).

For determination of absolute copy numbers, we used gene-specific standards amplified from neurosphere cDNA to generate standard curves. PCR standards for each gene of interest (GOI) were generated three times independently, each time from six pooled PCR reaction tubes after performing the RT-PCR (= 90 µl pooled samples of h and mNPCs each). The pooled samples were purified with QIAquick PCR purification kit (Qiagen, Hilden, Germany) and products were eluted with 50 µl EB buffer (included in the kit). The DNA concentration was measured using the NanoQuant plate device in the Tecan infinite M200 Pro reader and the number of cDNA molecules/µl was calculated. Stock solutions of 1.5×10^8 molecules/µl were generated and stored at -20°C. Standard curves were generated by running six times the same volume of cDNA (2.5 µl) with defined molecule numbers (1.5×10^2 up to 1.5×10^7 molecules/µl) as well as the samples with unknown gene copy numbers in the Rotor-Gene Q instrumentation (Qiagen, Hilden, Germany). From the standard curves the copy numbers of the GOI were calculated in the proliferating/differentiating NPC samples and normalized to the copy numbers of *β-actin*.

The ddCT was calculated as follows: First the dCT between the CT of the GOI and the corresponding *β-actin* CT was calculated for each sample. The dCT values of the triplicates

treated with solvent were pooled. The ddCT was calculated by subtraction of the sample dCT by the mean solvent control dCT. Then 2^{-ddCT} was calculated. Data was pooled for the three replicates of each treatment and the standard deviation was calculated.

As stated in the methods section, each experiment contained triplicates of 50 proliferating or triplicates of 10 differentiated neurospheres/treatment.

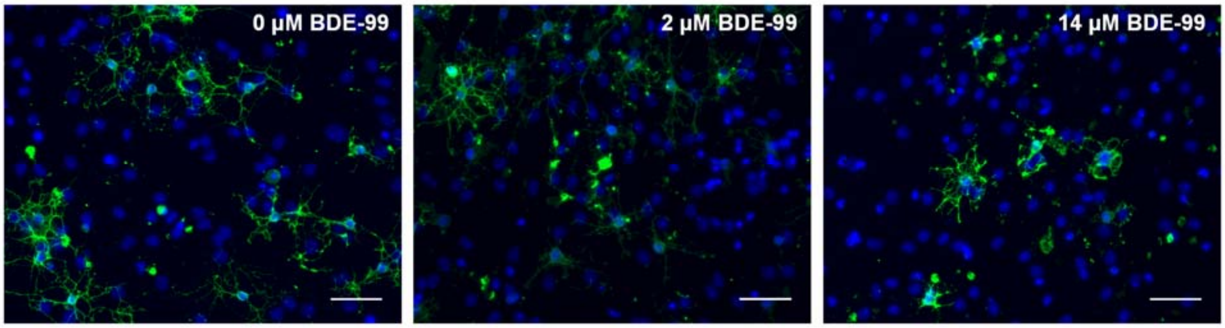

Fig. S1: Fluorescence microscope images of mouse O4$^+$ cells differentiated with BDE-99 treatment. Mouse NPCs were differentiated with DMSO, 2 µM or 14 µM BDE-99 (human or mouse IC50 concentration for reduction of formation of O4$^+$ cells, respectively) for 5 days. O4$^+$ cells were visualized by taking fluorescent images of O4 immunostained cells (green) and stained nuclei (blue) with Hoechst 33258 (scale bar: 50 µm).

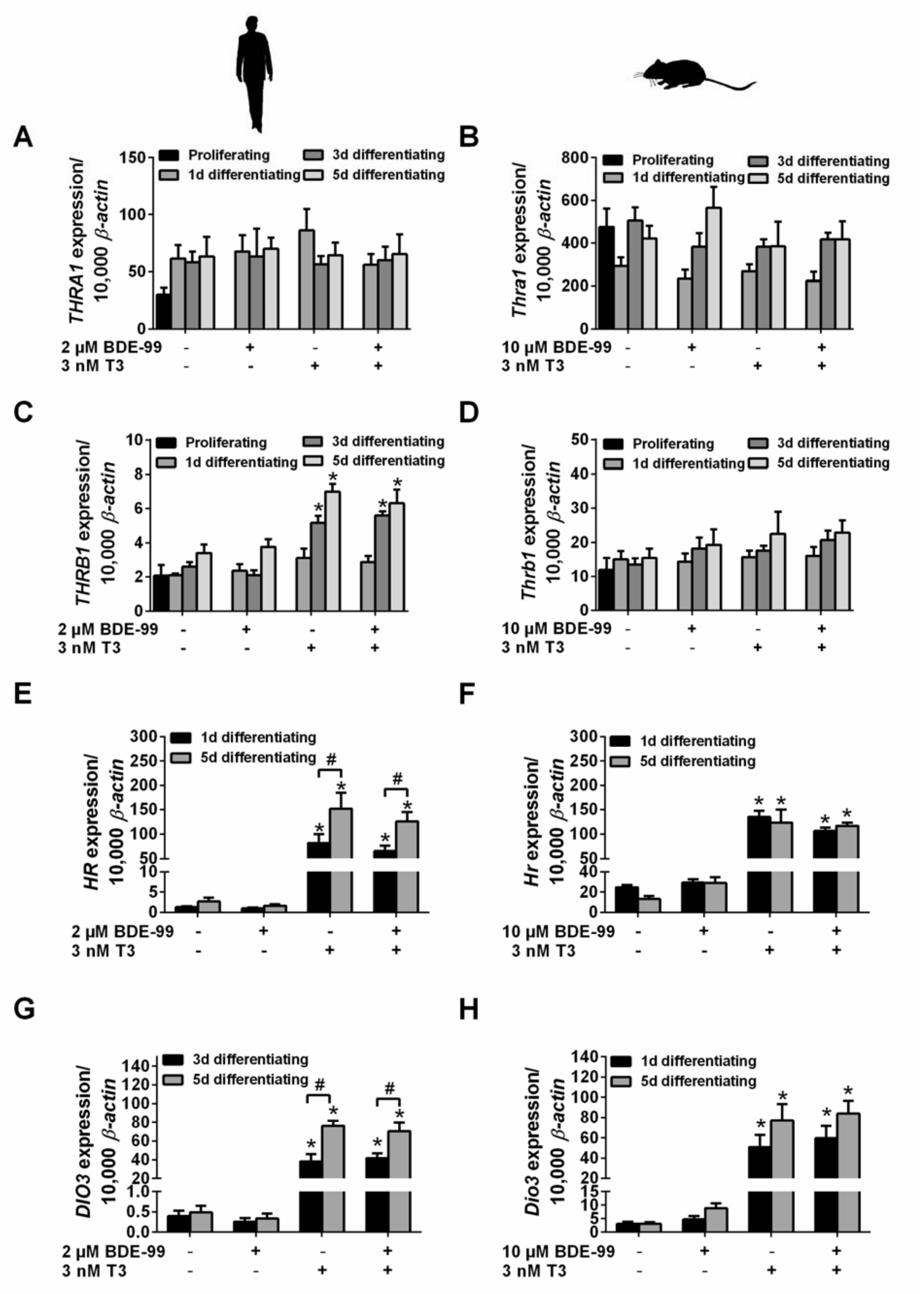
A
B
C
D
E
F
G
H
Proliferating
1d differentiating
3d differentiating
5d differentiating
THRA1 expression/ 10,000 β-actin
Thra1 expression/ 10,000 β-actin
THRB1 expression/ 10,000 β-actin
Thrb1 expression/ 10,000 β-actin
HR expression/ 10,000 β-actin
Hr expression/ 10,000 β-actin
DIO3 expression/ 10,000 β-actin
Dio3 expression/ 10,000 β-actin
2 µM BDE-99
10 µM BDE-99
3 nM T3

Fig. S2: Expression and functionality of TH signaling components. RT-PCR was performed for non-exposed proliferating or 1, 3 and/or 5 days in presence of solvents, BDE-99, T3 or respective co-exposures differentiated human (A,C,E,G) and murine (B,D,F,H) NPCs. Gene expression of thyroid hormone receptors: human A) THRA1 (n=5) and C) THRB1 (n=5) and murine B) Thra1 (n=4) and D) Thrb1 (n=4), (E+F) hairless (HR/Hr; n=4) and G+H) deiodinase 3 (DIO3/Dio3; n=3 (human), n=4 (mouse)) is shown as mean + SEM. Gene copy numbers are shown normalized to β-actin expression. $p<0.05$ was considered significantly different from the solvent control at the respective differentiation day (*) or from the same treatments at different differentiation days (#).

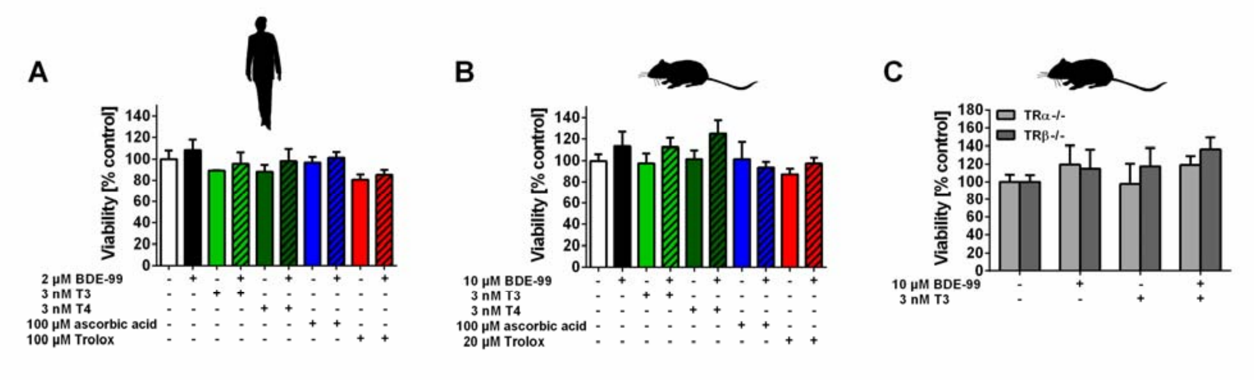

Fig. S3: Viability data for substance treatments in human, mouse wildtype and mouse TR knockout NPC experiments. Human (A) and mouse (B,C) NPCs were differentiated in presence of solvent(s) or compounds for 5 days as indicated above and an Alamar-Blue assay was performed two hours prior fixation from the same experiments for which formation of $O4^+$ cells is shown in the main manuscript (Fig. 2B,E; 4A; 5B,C,E,F). Data is shown as mean + SEM from at least three independent experiments. TwoWay ANOVA analysis with Tukey's post test did not reveal significant effects on viability ($p<0.05$).

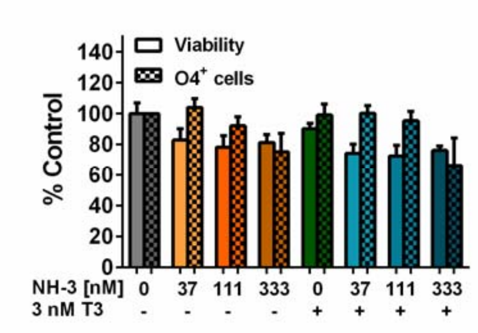

Fig. S4: Viability and formation of O4^{+} cells of NH-3 and/or T3 treated hNPCs. Human NPCs were differentiated in presence of solvent(s), NH-3 and/or T3 for 5 days and an Alamar-Blue assay was performed two hours prior fixation. Cells were stained with O4 antibody. Viability and formation of O4^{+}cells are shows as mean + SEM in % of control (n=3). TwoWay ANOVA analysis with Tukey's post test did not reveal significant NH-3 effects ($p<0.05$).

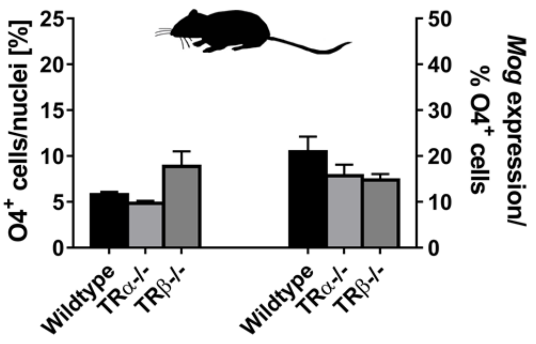

Fig. S5: Solvent control data for wildtype, TRα-/- and TRβ-/- mouse NPCs for oligodendrocyte formation and maturation. Mouse neurospheres of each genotype were treated with solvent for 5 days. Left part and left y-axis) Cells were fixed and $O4^+$ cells were immunocytochemically stained with O4 antibody and nuclei were counterstained with Hoechst 33258. The percentage of $O4^+$ cells/nuclei in the migration area is shown in %. Data are shown as mean + SEM (n=4 for each genotype). Right part and right y-axis) RNA was isolated, cDNA was transcribed and RT-PCR was performed. *Mog* expression was normalized to the expression of *β-actin* and was then divided by the percentage of differentiated $O4^+$ cells in the neurosphere migration area. Data are shown as mean + SEM, n=3 (wildtype), n=4 (knockout). Data of knockout mice were compared to wildtype data with student t-test using Welsh's correction. No statistical differences in $O4^+$ cells formation or oligodendrocyte maturation could be observed between the knockout and wildtype NPCs ($p<0.05$).

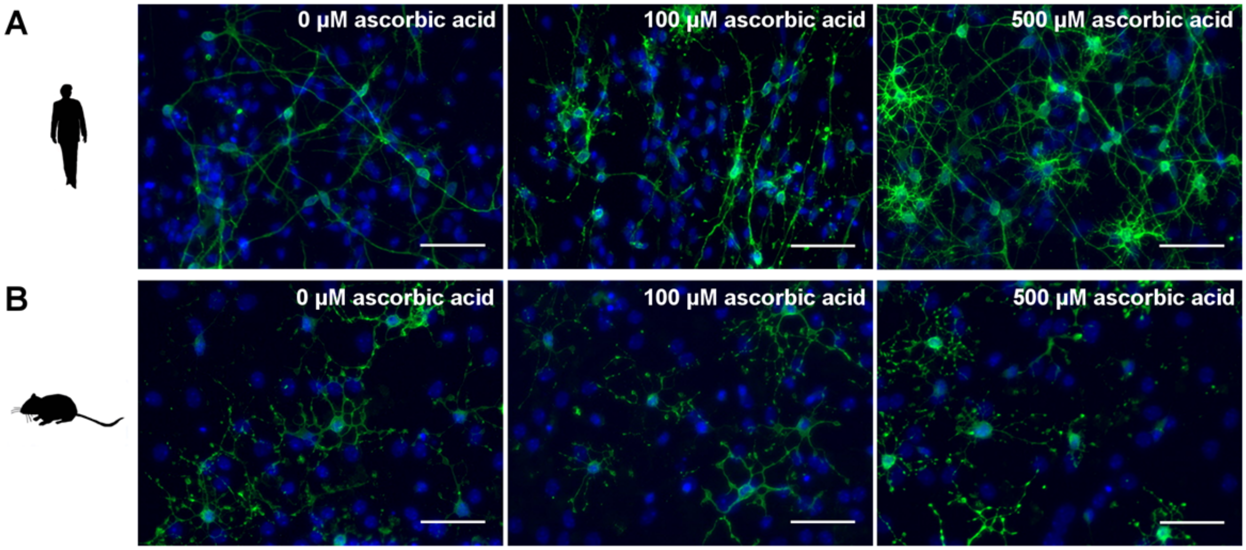

Fig. S6: Ascorbic acid effects on formation and maturation of $O4^+$ cells from human and mouse NPCs.

Human (A) and mouse (B) NPCs were differentiated in presence of water, 100 µM or 500 µM ascorbic acid for 5 days and cells were fixed. $O4^+$ cells were immunocytochemically stained with O4 antibody and nuclei were counterstained with Hoechst 33258. Representative fluorescence microscope pictures of human (A) and murine (B) $O4^+$ cells differentiated with water, 100 µM or 500 µM ascorbic acid (scale bar: 50 µm).

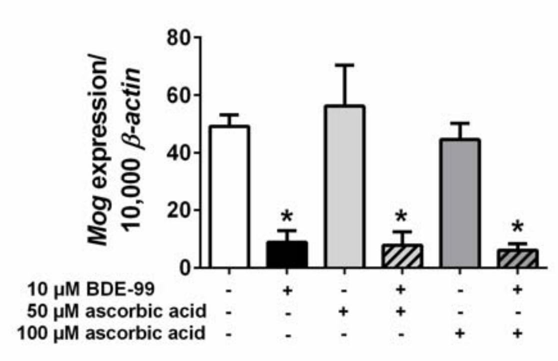

Fig. S7: m*Mog* expression after mNPC treatment with BDE-99 and/or ascorbic acid. Murine NPCs were differentiated in presence of solvent(s), BDE-99 and/or ascorbic acid for 5 days and m*Mog* expression was studied by RT-PCR. m*Mog* expression is shown normalized to β-actin as mean + SEM (n=3). TwoWay ANOVA analysis with Tukey's post test was performed. $p<0.05$ was considered significantly different from the solvent control (*).

Table 1: Primer sequences.

	Species	Forward primer	Reverse primer
β-actin	human	CAGGAAGTCCCTTGCCATCC	ACCAAAAGCCTTCATACATCTCA
	mouse	CACCCGCCACCAGTTCGCCA	AGCACAGGGTGCTCCTCAGGG
catalase	human (*CAT*)	CGTGCTGAATGAGGAACAGA	AGTCAGGGTGGACCTCAGTG
	mouse (*Cat*)	ATGACAACCAGGGTGGTGCCC	GCGGGCCCCATAGTCAGGGT
deiodinase	human (*DIO3*)	AAGTGCCCAGACCTTCCAAA	AAAGAAACCCTTGCAGCCTTC
	mouse (*Dio3*)	ATCCGTGTTTTCCCTCTCGTC	GCTGGAAGGCCATTTTCTGTC
glutathione peroxidase	human (*GPX*)	TCTGTTGCTCGTAGCTGCTGC	GGGGTCAAGAGGAGGAGAGA
	mouse (*Gpx*)	TCGGACACCAGGAGAATGGCAA	GCGGCACACCGGAGACCAAA
hairless	human (*HR*)	CCGCTTTCTCCAGATGGTGTG	AGAGGAAGTGCTGAGTGACG
	mouse (*Hr*)	AATACTGTGCCCACCAAGGG	TGAGTTCACACCATAGGCCG
heme oxygenase-1	human (*HMOX1*)	GCCATGAACTTTGTCCGGTG	GGATGTGCTTTTCGTTGGGG
	mouse (*Hmox1*)	CTCTGTCCAATGTGGCCTTCT	CACTGGCTGGATGTGCTTTTG
myelin basic protein	human (*MBP*)	CAGAGCGTCCGACTATAAATCG	GGTGGGTTTTCAGCGTCTA
myelin oligodendrocyte glycoprotein	mouse (*Mog*)	TCCATCGGACTTTTGATCCTCA	CGCTCCAGGAAGACACAACC
superoxide dismutase	human (*SOD*)	GGCCGATGTGTCTATTGAAGA	GGGCCTCAGACTACATCCAA
	mouse (*Sod*)	TCGAGCAGAAGGCAAGCGGTG	TCTCTTCATCCGCCGGGCCA
thyroid hormone receptor α1	human (*THRA1*)	GCTGCTAATGTCAACAGA	CCCCGATCATGCGGAGGTCA
	mouse (*Thra1*)	GCGAAAATTCCTGCCGGATG	GATCTGGTCTTCGCAAGGCA
thyroid hormone receptor β1	human (*THRB1*)	AAGTGCCCAGACCTTCCAAA	AAAGAAACCCTTGCAGCCTTC
	mouse (*Thrb1*)	ATCCGTGTTTTCCCTCTCGTC	GCTGGAAGGCCATTTTCTGTC
